# Influence of Maqian essential oil on gut microbiota and immunoresponses in type 1 diabetes: In silico study

**DOI:** 10.1016/j.heliyon.2024.e29490

**Published:** 2024-04-15

**Authors:** Mahmoud Dahab, Hajo Idris, Ping Zhang, Mohammed Aladhadh, Eid A. Alatawi, Long Chiau Ming, Khang Wen Goh, Hooi-Leng Ser

**Affiliations:** a Department of Microbiology, Faculty of Pure and Applied Sciences, International University of Africa, P.O. Box 2469, Khartoum, Sudan; b Department of Physics, College of Science, Imam Mohammad Ibn Saud Islamic University (IMSIU), Riyadh, 11623, Saudi Arabia; c Center for Integrative Conservation, Yunnan Key Laboratory for the Conservation of Tropical Rainforests and Asian Elephants, Xishuangbanna Tropical Botanical Garden, Chinese Academy of Sciences, Mengla, 666303, China; d Department of Food Science and Human Nutrition, College of Agriculture and Food, Qassim University, Buraydah 51452, Saudi Arabia; e Department of Medical Laboratory Technology, Faculty of Applied Medical Sciences, University of Tabuk, 71491, Saudi Arabia; f Department of Medical Sciences, School of Medical and Life Sciences, Sunway University, Sunway City 47500, Malaysia; g Faculty of Data Science and Information Technology, INTI International University, Nilai, Malaysia; h Department of Biological Sciences, School of Medical and Life Sciences, Sunway University, Sunway City, 47500, Malaysia

**Keywords:** Maqian essential oil, Gut microbiota homeostasis, Inflammation, In silico, Human and disease, Wellbeing

## Abstract

Diversity and homeostasis of gut bacterial composition is highly associated with the pathogenesis of insulin dysfunction and type 1 diabetes melittus (T1D), hence emerged in parallel with the activation of autoimmunity. We aimed to study the bioactive potential of essential oil from *Zanthoxylum myriacanthum* var. pubescens Huang (Maqian) through computational approaches. Twelve chemical constituents derived from Maqian essential oil were docked with selected proteins (i.e., 3pig, 1kho, 7dmq, 4m4d, 2z65, 4glp, and 3fxi) in which are involved in gut microbiota modulation in T1D. Subsequently, the prediction of bioavailability properties of the small molecules were evaluated. Among all chemical constituents, the post-docking interaction analysis demonstrated that α-phellandrene exhibits the strongest binding affinity and induces gut microbiota modulation with β-fructofuranosidase from *Bifidobacterium longum*. The current result revealed the potential of 3-Carene and α-Pinene in inducing specific changes in gut microbiota downregulating *Clostridium perfringens* and quenching *Leptotrichia shahii* respectively. β-Pinene possess exceptionally strong binding affinity that effectively disrupt the interaction between lipopolysaccharide and its cognate receptors, while α-Phellandrene was exhibited the uppermost binding affinity with TLR4/MD2 and could likely target TLR4 stimulating lipopolysaccharide. Our results are the first to report on the gut microbiota modulation effects of α-Phellandrene and β-Phellandrene via actions on LPS binding to CD14 and the TLR4 co-receptor signaling. In conclusion, our findings based on computational approaches, small molecules from Maqian present as promising agents which could regulate inflammatory response and modulate gut microbiota in type 1 diabetes mellitus.

## 1 Introduction

Accumulated evidence indicated that the dysregulation of gut microbiota (GM) is closely involved in the pathogenesis of Type 1 diabetes mellitus (T1D) and it is related inflammation pathways [1]. Modulation of GM could be a valuable approach for T1D treatment with target different pathways and maintaining blood glucose as ultimate goal. However, most of immunomodulatory showed only diffident accomplishment in clinical studies, their effects on GM have not been known yet.

The GM is presented as a diverse and dynamic community of microorganisms that inhabits the gastrointestinal tract; it plays a pivotal role in human health by modulating the innate and adaptive immune systems and altering intestinal permeability [2]. Commensal bacteria, which are the major type of bacteria residing in the gastrointestinal tract, perform essential biological functions that are beneficial for their hosts' health [2,3]. Dysbiosis, an imbalance in the ecosystem of commensal species, can disrupt host homeostasis and their metabolic pathways, leading to other negative consequences for human health [4].

Bifidobacteria are anaerobic Gram-positive bacteria that predominate in the human gut. They are considered to be major targets for GM modulation and probiotics in the prevention and treatment of gastrointestinal disorders [5], as well as promising agents in the treatment of Type 2 diabetes [6]. *Bifidobacterium longum*, a major bifidobacteria, usually dominating bacteria in the infants [7], It has been shown to be crucial for protective and anti-inflammatory properties [8,9], even though studies on the use of medicinal plants involving this microbe to treat inflammation are still lacking. Similarly, previous studies have revealed that changes in the *Clostridium perfringens* in the gut are associated with compromised gut integrity and increased risk of T1D [[10], [11], [12]]. *Leptotrichia* spp. has been closely linked to autoimmune diseases and inflammatory bowel diseases (IBDs) [13,14]. The potential roles of phytochemical constituents to modulate *Clostridium* spp. and *Leptotrichia* spp. in the progress of GM still necessitating further research.

Even small amounts of lipopolysaccharide (LPS), a component of the outer membrane of Gram-negative bacteria [15], can activate the innate immune response, leading to dysregulation and increases of proinflammatory cytokine production. This uncontrolled inflammatory response can impair pancreatic beta-cell function [16,17]. LPS, a bacterial toxin glycan component, is a virulence factor and primary inflammatory pathogen [18]. Increasing evidence from mouse models suggests that LPS-induced inflammation is a key factor in T1D [19,20]. The GM is a major source of LPS, and dysbiosis can impair the gut barrier, allowing LPS to translocate into the bloodstream [21]. Therefore, in recent years, there has been growing interest in elucidating the molecular mechanisms underlying LPS-induced intestinal inflammation in T1D, with the aim of developing new therapeutic strategies.

Toll-like receptor 4 (TLR4) plays a pivotal role in defending the host from exogenous and endogenous exposures, including infectious microbes [22,23]. TLR4 initiates an immediate response to bacterial infection [24]. Additionally, GM alteration can disrupt the intestinal mucosal barrier, leading to the leakage of LPS and fatty acids, which further activates TLR4 and results in metabolic inflammation [25]. Henceforth, TLR4 therapy may be a promising and unique immunomodulatory approach to treating gut inflammation and T1D, providing an alternative anti-inflammatory strategy. Upon binding to TLR4, LPS triggers a cascade of signaling events that culminates in the production of pro-inflammatory mediators, orchestrating the host's immune response against assaulting pathogens [26]. Cluster differentiation 14 (CD14) is a multi-functional co-receptor expressed on the plasma membrane of most myeloid cells [27]. It enhances innate immune responses by facilitating LPS recognition by the TLR4-CD14 complex [28,29].

*Zanthoxylum* species and their phytochemical constituents are widely used as medicines and spices. They have been reported as potential sources of anti-inflammatory, antibacterial, antioxidant, anti-cancer, antimalarial, and antisickling drugs [[30], [31], [32], [33], [34], [35]]. *Zanthoxylum myriacanthum* var. pubescens Huang, a popular medicinal plant in China, is known as Maqian. The fruits of Maqian have been traditionally used to treat inflammation, detoxification, analgesia, digestive disorders, and pain [36]. Targeting different proteins involved in gut inflammation by small molecules from medicinal plants might be a potential and important strategy for response to GM modulation in T1D.

Acknowledging limitations in conventional GM homeostasis methods underscores the imperative need for alternative strategies, leading to the exploration of Maqian compounds as natural antibacterial, antiinflammatory, and antidiabetic agents against gastrointestinal disorders. The current study aimed to identify the structure and function relationship of phytochemical compounds derived from Maqian essential oil (MQEO), by using computational approaches of these compounds with different target proteins (i.e., β-fructofuranosidase, *C. perfringens* α-toxin, Cas13a anti-tag RNA ternary, LPS-binding protein, TLR4/MD2, and human CD14) which are involved in GM modulation in T1D. The outcomes of this computational study would serve as a benchmark for identifying, evaluating particular plant-based inhibitors, opening up new possibilities for the creation of cutting-edge, induced microbiota modulation and more potent anti-inflammatory drugs.

## 2 Materials and methods

### 2.1 Retrieval and refining target receptor proteins

Seven receptor structures, β-fructofuranosidase from *B. longum* (PDB: 3pig), *C. perfringens* α-toxin, (PDB: 1kho), Cas13a anti-tag RNA ternary, *Leptotrichia shahii,* (PDB: 7dmq), LPS-binding protein (LBP) (PDB: 4m4d), TLR4/MD2 (PDB: 2z65), human CD14 (PDB: 4glp), and TLR4/MD-2 (PDB: 3fxi), were obtained from the RCSB protein data bank (https://www.rcsb.org/). The dock preparation was performed for all proteins, and we prepared proteins structure by deleting ligands, water molecules, adding polar hydrogen atoms, as well as other heteroatoms using UCSF Chimera X software version 1.6.1. The improved PDB file was then utilized to simulate docking.

### 2.2 Ligands preparation

In a previous study by our research group, the chemical compositions of MQEO were investigated [36], and several components were elucidated *in vitro* and *in vivo*. For the current study, the targeted twelve MQEO phytochemical compounds were retrieved from the PubChem-NCBI database (https://pubchem.ncbi.nlm.nih.gov/) in structure data file (SDF) format. The compounds included α-Pinene (CID: 6654), β-Pinene (CID: 14896), α-Phellandrene (CID: 7460), 3-Carene (CID: 26049), *p*-Cymene (CID: 7463), D-Limonene (CID: 440917), β-Phellandrene (CID: 405237423), *cis*-β-Ocimene (CID: 249959750), *trans*-β-Ocimene (CID: 249959748), α-Terpineol (CID: 253657446), n-Decanal (CID: 8175), and Acetic acid octyl ester (CID: 8164). To obtain optimized chemical structures and minimized energy, the ligand structures were processed using Avogadro software version 1.2, with the specified Merck Molecular Force Field (MMFF), 1000 steps, the Conjugate Gradient algorithm, and a convergence criterion of 10⁻⁶. The optimized chemical structures were saved in MOL2 format for further analysis.

### 2.3 Molecular docking

The molecular docking was performed using the CB-Dock 2 web server, a powerful and user-friendly platform for protein-ligand docking analysis (https://cadd.labshare.cn/cb-dock2/). The target macromolecules (receptors) and the corresponding small molecules (ligands) were uploaded to the CB-Dock 2 web interface in their respective file formats. The web tool's default parameters were used, and the server was initiated the docking process, employing a combination of three key algorithms: cavity detection, docking, and template-based fitting. Algorithm utilizes AutoDock Vina, a well-established docking tool, to optimize the ligand's conformation and position within the binding pocket. The resulting binding poses were then evaluated based on their binding affinities and visualized using Discovery Studio Visualizer version 21.1.0.20298 and UCSF Chimera X software, and 2D and 3D images were generated.

### 2.4 Evaluation of screened metabolite's small molecules profile

After the docking simulation was finished, the identified compounds in the simplified molecular-input line-entry system (SMILES) format were exported to Swiss ADME web server. The Swiss ADME webserver (www.swissadme.ch) was utilized to screen metabolites, assess the drug-like properties, and bioavailability of twelve compounds derived from MQEO.

## 3 Results

### 3.1 Molecular docking analysis of screened MQEO small molecules

**Fig. 1:**
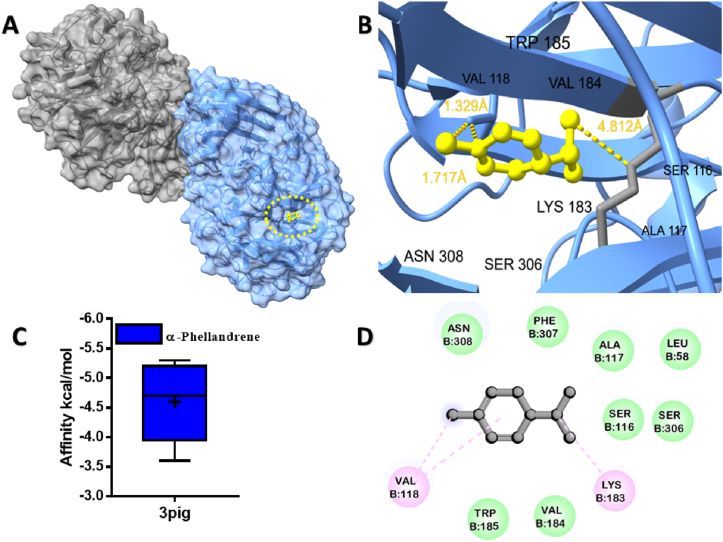
Molecular docking analyses of α-Phellandrene against β-fructofuranosidase proteins of *Bifidobacterium longum*. (**A**), Pose view of the interaction of α-Phellandrene to the β-fructofuranosidase protein, (**B**) 3D interaction of α-Phellandrene with β-fructofuranosidase protein, (**C**) Box plot depicted binding affinity scores for predictions of α-Phellandrene (blue) and with β-fructofuranosidase (3pig) protein, (**D**) 2D interaction of α-Phellandrene with key residues. (For interpretation of the references to colour in this figure legend, the reader is referred to the Web version of this article.)

The molecular docking analysis demonstrated the potential binding affinity of MQEO towards proteins, as evidenced by the docking scores presented in (Table 1). A higher negative docking score indicates a stronger binding affinity. Among the analyzed compounds, α-Phellandrene exhibited the most favorable binding affinity of −5.3 kcal/mol with β-fructofuranosidase from *B. longum* (Fig. 1). The alkyl/pi-alkyl interactions of α-Phellandrene with the Lys 183, and Val 118 residues of B chain are depicted in (Fig. 1B).

**Table 1:** Binding energy (kcal/mol) of MQEO small molecules with target proteins.

No	Compound name	3pig^a^	1kho^b^	4m4d^c^	2z65^d^	4glp^e^
1.	α-Pinene	−5.2	−4.2	−6.5	−7.2	−5.1
2.	β-Pinene	−5	−4.3	−7.2	−7	−5.3
3.	α-Phellandrene	−5.3	−4.3	−6.6	−7.5	−5.8
4.	3-Carene	−5.2	−4.5	−5.1	−7.2	−5.6
5.	P-Cymene	−5.2	−4.4	−7.1	−7.1	−5.7
6.	D-Limonene	−5	−4.2	−6.4	−6.8	−5.5
7.	β-Phellandrene	−5.2	−4.2	−6.8	−7.2	−6.0
8.	*cis*-β-Ocimene	−4.9	−4.4	−6.5	−5.9	−5.9
9.	*trans*-β-Ocimene	−4.8	−4.2	−6.3	−6	−5.8
10.	α-Terpineol	−5.1	−4.2	−6.2	−6.7	−5.2
11.	n-Decanal	−4.3	−3.6	−5.4	−5.4	−5
12.	Acetic acid octyl ester	−4.6	−3.2	−5.3	−5.3	−5.2

a β-fructofuranosidase from *Bifidobacterium longum*.

b *Clostridium perfringens* α-toxin.

c LPS-binding protein.

d TLR4/MD2.

e Human CD14.

**Fig. 2:**
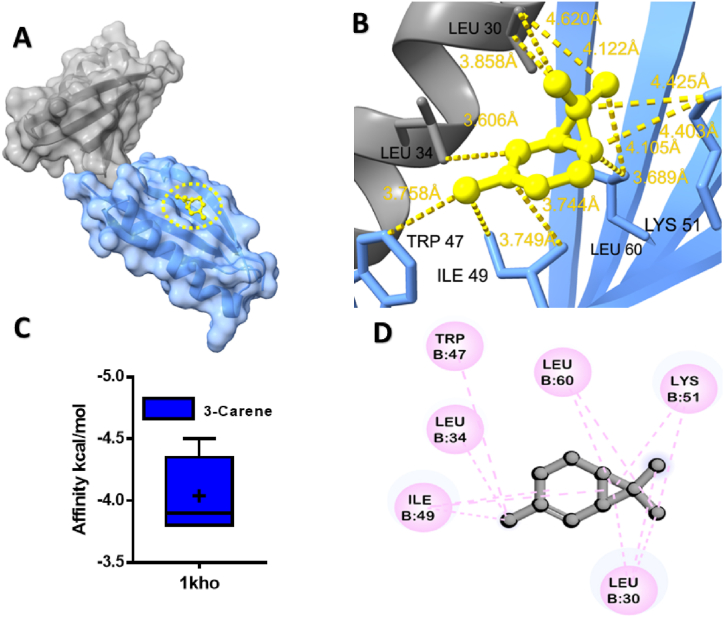
Molecular docking analyses of 3-Carene against α-toxin proteins of *Clostridium perfringens*. (**A**), Pose view of the interaction of 3-Carene to the α-toxin protein, (**B**) 3D interaction of 3-Carene with protein, (**C**) Box plot depicted binding affinity scores for predictions of 3-Carene (blue) and with α-toxin (1kho) protein, (**D**) 2D interaction of α-toxin with key residues. (For interpretation of the references to colour in this figure legend, the reader is referred to the Web version of this article.)

Additionally, the major constituents of MQEO were computationally verified to interact with the target protein of *C. perfringens* α-toxin (Fig. 2A), the best docked pose of 3-Carene revealed alkyl and pi-alkyl interactions with the Leu 30, Leu 34, Leu 60, Lys 51, Trp 47, and Ile 49 residues of the 1kho protein (Fig. 2C). These findings suggest that MQEO and its constituents possess the potential to modulate *B. longum* and *C. perfringens* homeostasis through direct binding interactions.

**Fig. 3:**
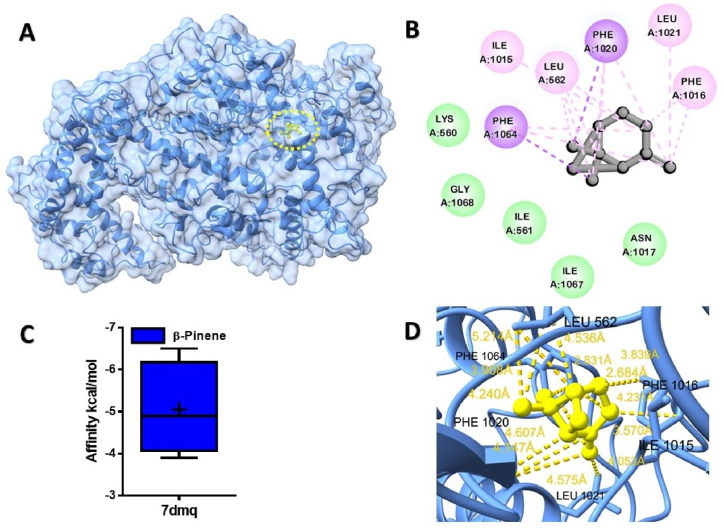
Molecular docking analyses of β-Pinene against Cas13a proteins of *Leptotrichia shahii*. (**A**), Pose view of the interaction of α-Pinene to the Cas13a protein, (**B**) 3D interaction of α-Pinene with protein, (**C**) Box plot depicted binding affinity scores for predictions of α-Pinene (blue) and with Cas13a (7dmq) protein, (**D**) 2D interaction of Cas13a with key residues. (For interpretation of the references to colour in this figure legend, the reader is referred to the Web version of this article.)

**Fig. 4:**
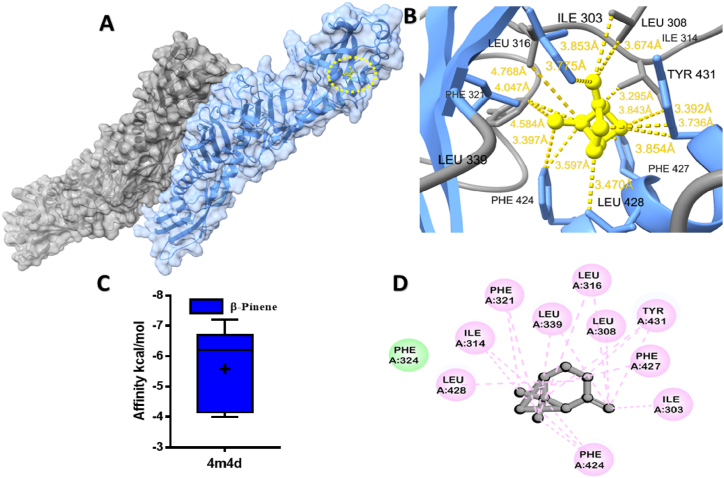
Molecular docking analyses of β-Pinene against LPS-binding protein. (**A**), Pose view of the interaction of β-Pinene to the LPS-binding protein, (**B**) 3D interaction of β-Pinene with LPS-binding protein, (**C**) Box plot depicted binding affinity scores for predictions of β-Pinene (blue) and with LPS-binding protein (4m4d) protein, (**D**) 2D interaction of LPS-binding protein with key residues (For interpretation of the references to colour in this figure legend, the reader is referred to the Web version of this article.)

The best docked pose of β-Pinene displayed alkyl pi-alkyl, and pi-Sigma interactions with amino acid residues Phe 1016, Phe 1020, Phe 1064, Leu 1021, Leu 562, Ile 1015, and Lys 560 protein of 7dmq from *Leptotrichia shahii* (Fig. 3C). Similarly, β-Pinene has shown most favorable binding affinity toward LPS of A chain LPS-binding protein (Fig. 4A).

**Fig. 5:**
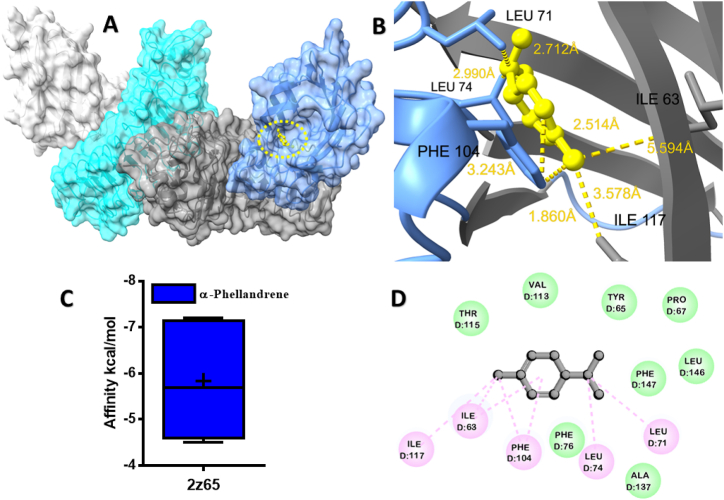
Molecular docking analyses of α-Phellandrene against TLR4/MD2. (**A**), Pose view of the interaction of α-Phellandrene to TLR4/MD2, (**B**) 3D interaction of α-Phellandrene with TLR4/MD2, (**C**) Box plot depicted binding affinity scores for predictions of α-Phellandrene (blue) and with TLR4/MD2 (2z65), (**D**) 2D interaction of α-Phellandrene with key residues. (For interpretation of the references to colour in this figure legend, the reader is referred to the Web version of this article.)

**Fig. 6:**
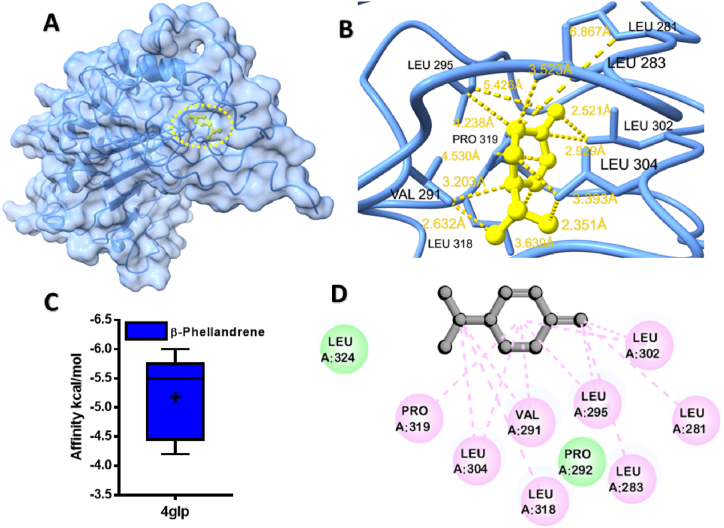
Molecular docking analyses of β-Phellandrene against human CD14. (**A**), Pose view of the interaction of β-Phellandrene to the human CD14, (**B**) 3D interaction of β-Phellandrene with human CD14, (**C**) Box plot depicted binding affinity scores for predictions of β-Phellandrene (blue) and with human CD14 (4glp), (**D**) 2D interaction of human CD14 with key residues. (For interpretation of the references to colour in this figure legend, the reader is referred to the Web version of this article.)

Among MQEO constituents, the highest binding affinity was unveiled for α-Phellandrene with TLR4 protein (−7.5 kcal/mol) (Fig. 5A). β-Phellandrene interaction with the cluster differentiation 14 showed non-conventional hydrogen bonds with Leu 281, Leu 283, Leu 295, Leu 302, Leu 304, Leu 318, Val 291, and Pro 319 of B chain (Fig. 6B). Interestingly, α-Pinene and β-Pinene also yielded good binding affinity of −7.2 kcal/mol in the case of TLR4/MD-2 protein (Fig. S1) and (Table S1).

### 3.2 In silico prediction of metabolite's small molecules profile

**Fig. 7:**
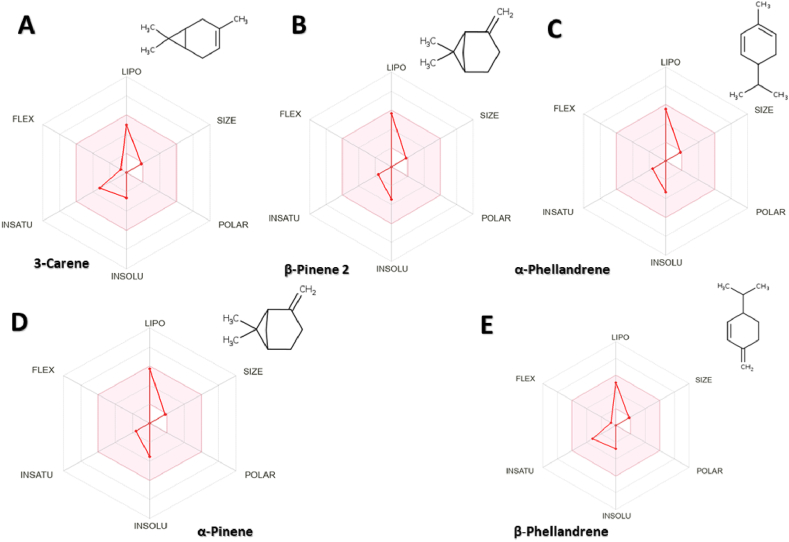
Evaluation of bioavailability of the MQEO, a small molecules with highest binding affinity modulete gut microbiota. The pink area of the bioavailability radar graph represents drug-likeness properties of the molecule. (For interpretation of the references to colour in this figure legend, the reader is referred to the Web version of this article.)

The results revealed that all MQEO compounds exhibited favorable physicochemical properties and were situated within the pink region of the Bioavailability Radar graph (Fig. 7 and S2), indicating high oral bioavailability potential. These findings suggest that MQEO compounds possess promising drug-like characteristics and warrant further investigation of MQEO compounds as promising therapeutic agents.

## 4 Discussion

Traditional plants medicine has long been actively investigated in treating DM and their associated complications, because they are considered to have little toxicity and thus suitable for long term use. In other hand, plant that containing terpenes and their oxygenated compounds are identified ingredients with the efficacy in maintains GM homeostasis and to ameliorate T1D associated symptoms [37]. Type 1 Diabetes has been associated with severe dysbiosis and increased intestinal permeability. In particular, an increase in Bacteroides and *Clostridium* spp. along with a decrease in bifidobacteria and lactobacilli have been found in patients [10,38,39]. However, our group previously shown that MQEO had protective effect against intestinal inflammation *in vivo* and *in vitro* with potent anti-inflammatory properties via suppression of TNF-α and IL-1β [40].

Essential oils (EOs), a volatile compound from aromatic plants, process a wide range of biological activities. There is growing evidence showing anti-diabetic effects of EOs extracted from various sources such as *Melissa officinalis, Lavandula stoechas* L [41]. and *Pinus koraiensis* [42]. Essential oil molecules are capable of selectively targeting some bacterial species and leaving unaltered the bacterial populations considered healthy [43]. The fruits from Maqian are used by local people not only as a spice for roasting meat and boiling fish, but also as a remedy for digestive disorders. The composition of maqian essential oil (MQEO) is encompassed by several terpenes with limonene, α-pinene, β-myrcene, and phelladrene as major phytochemical components [44]. In recent year, MQEO constituents demonstrated therapeutic possible as propitious bioactive compounds for developing new anti-cholera agents through computational approaches [45]. In a previous study, we showed that oral administration of MQEO protected DSS-induced colitis in mice through suppression of inflammatory cytokines and NF-κB activation [40]. In parallel study, we have shown that the increased expression of Nrf2 in renal tissue of T1D mice is at least one mechanism for the renoprotective effect of MQEO [44]. Furthermore, our group revealed that MQEO enhances liver function by normalizing blood glucose level, elevating antioxidant, and to have hepatoprotective effect on in streptozotocin induced T1D in mouse [46]. Based to our previous work on MQEO, we hypothesized that MQEO might also has immunomodulatory effect by directly targeting GM in T1D disease.

The GM has been implicated in the pathogenesis of various inflammatory diseases, extending beyond intestinal disorders to encompass neurological, hepatic, pulmonary, and musculoskeletal disorders. While strong correlations have been established between specific microbial signatures and inflammation, the precise role of these microbes as disease markers or disease drivers remains an area of active exploration [47]. A substantial body of evidence supports a strong connection between GM dysbiosis and the development of inflammatory disorders. This association has been extensively documented across various pathological situations, including T1D [10] and inflammatory bowel disease (IBD) [48]. These findings suggest that alterations in the gut microbial community can play a significant role in initiating or exacerbating inflammatory processes. Maffeis et al. identified three specific bacteria, *Dialister invisus*, *Gemella sanguinis*, and *B. longum*, that were associated with altered intestinal permeability in individuals with T1D. Moreover, they observed a consistent trend of reduced *Bifidobacterium* spp. richness in T1D patients compared to healthy controls [12].

Recent studies have demonstrated that supplementation with a combination of EOs can significantly enhance bacterial diversity and promote the growth of beneficial probiotic bacteria, particularly *Streptococcus* spp. and *Bifidobacterium* spp., within the caecal microbiota. This suggests that EOs could potentially serve as therapeutic agents for modulating the GM and promoting gut health [49]. Our finding demonstrates that α-phellandrene exhibits favorable binding affinities and forms stable 2D and 3D interactions with β-fructofuranosidase from *B. longum*, a protein that plays a crucial role in maintaining gut microbial homeostasis. These interactions contribute to the overall binding energy of α-phellandrene, potentially explaining its ability to directly modulate Bifidobacterium and intestinal endotoxin levels.

*Clostridiaceae* is one of the main families of the Firmicutes phylum that inhabit the tract [50]. Li et al. demonstrated that carvacrol and thymol could effectively modulate the GM of broilers, leading to a reduction in the populations of *C. perfringens* and *Escherichia coli* [51]. In a prospective study, an increase in microbial diversity and abundance in *Ruminococcus* spp. and *C. perfringens* was detected [52]. Here, we reveal that 3-Carene and α-Pinene possess the ability to modulate the composition of the GM by specifically targeting *C. perfringens* and *Leptotrichia shahii*, respectively.

Progressive disruptions in the GM can induce alterations in microbial metabolic pathways, leading to an enhanced production of toxins. These toxins, in turn, can activate the innate immune system, culminating in chronic low-grade intestinal inflammation [53]. Among the various molecular factors influencing the GM, LPS, a bacterial endotoxin stands out as a primary inducer of inflammation [54]. LPS may serve as a crucial molecular bridge connecting the GM, pancreatic inflammation, and the heightened susceptibility of islet macrophages to GM signals, potentially accelerating the development of T1D [55]. Furthermore, initiation of the innate immune response involves the coordinated interaction of several key proteins, including LBP, CD14, and TLR4 in conjunction with myeloid differentiation factor 2 (MD-2). The formation of the LPS/TLR4/MD-2 complex triggers a cascade of intracellular signaling events, leading to the activation of nuclear factor-κB (NF-κB) and the production of pro-inflammatory cytokines. These cytokines, in turn, orchestrate the recruitment and activation of additional immune cells, ultimately mounting a robust inflammatory response to shield the invading bacteria [56,57]. A notable characteristic shared by individuals with metabolic disorders is the higher proportion of Gram-negative bacteria, which coincides with elevated levels of LPS, which leads to systemic inflammation mediated by proinflammatory cytokines [58,59].

The present study delved into the intricate molecular mechanisms underlying receptor recognition and ligand-TLR interactions through the application of molecular docking approach. Computational protein-ligand docking methods hold immense promise for the development of novel small molecules targeting TLR4-related disorders [60]. As a matter of fact, GM can trigger different TLRs to induce both pro-diabetogenic and anti-diabetogenic signals. By simulating the interactions between ligands and TLR4, these methods can effectively identify, characterize potential drug candidates, and guide the design of highly specific and potent therapeutics capable of modulating TLR4 signaling and alleviating symptoms associated with TLR4-mediated diseases. The relationship between TLR4 signaling and the nuclear factor erythroid 2- related factor (Nrf2) pathway is of critical importance in the restoration of intrinsic anti-inflammatory defenses and tissue homeostasis following inflammation [61,62]. Our previous research demonstrated that the Nrf2/heme oxygenase-1 (HO-1) pathway acts as a potent antioxidant defense mechanism [44]. Molecular docking revealed that β-pinene possesses the ability to impede LPS-induced intestinal inflammation. These results suggest that the small molecules derived from MQEO possess exceptionally strong binding affinities that effectively disrupt the interaction between LPS and its cognate receptors, CD14, and the TLR4/MD-2 complex. This interference with LPS binding ultimately leads to the inhibition of TLR4 activation, thereby mitigating inflammatory responses and promoting intestinal homeostasis.

Understanding the functional implications of the binding energy variations could lead to the identification of novel therapeutic targets and strategies for the treatment of inflammatory diseases. Toll-like receptors serve as sentinels of the innate immune system, recognizing an array of PAMPs, including LPS [63,64]. LPS is transferred from CD14 to MD-2 through an interaction with the TLR4-LRR15 domain and the subsequent activation of innate immune responses [65]. In this study, protein-ligand docking provides compelling evidence that MQEO small molecules effectively inhibit the binding of LPS to its cognate receptors, CD14, and the TLR4/MD-2 complex. This interference with LPS binding ultimately leads to the inhibition of TLR4 activation, thereby mitigating inflammatory responses and promoting intestinal homeostasis. The strong binding affinity of β-pinene to LPS highlights its potential as a therapeutic agent for treating LPS-induced intestinal inflammation. Further research is necessary to validate findings, elucidate the mechanistic underpinnings of microbiota-mediated inflammation, determine whether specific microbial communities can be targeted therapeutically to alleviate inflammatory diseases, and the functional consequences of the observed binding energy differences between TLR4/MD-2 and human CD14 proteins would provide valuable insights into their roles in various biological processes.

Traditional single-molecule approaches may fall short in addressing the multifaceted nature of DM, this opens avenues for further research on plant-based interventions, aiming to unlock the therapeutic potential of nature's intricate chemical compositions beyond single-molecule limitations. In contrast, this study highlights the promise of a multi-target strategy for gut microbiota homeostasis. By harnessing the synergistic potential of various bioactive compounds found in Maqian, we could potentially mitigate diverse aspects of GM pathology, including gut inflammation, and intestinal endotoxin. We employed docking simulations to elucidate the predicted interactions between specific bioactive compounds from Maqian and relevant protein targets associated with GM modulation. This study, for the first time, unveiled the beneficial effects of twelve phytochemical constituents of Maqian, demonstrating their ability to interact with specific proteins at the molecular level. These findings suggest that the small molecules derived from MQEO could potentially modulate the composition of the GM, this modulation, in turn, could lead to a cascade of beneficial effects, including the amelioration of dysbiosis, the stimulation of immune responses, and the attenuation of T1D development. Additionally, our results indicate that MQEO and its constituents acts via surface receptors to activate downstream signaling pathways, further supporting its potential role in restoring internal anti-inflammatory defense mechanisms.

## 5 Conclusions

In conclusion, developing therapeutic strategies modulating gut microbiota remains a crucial unmet need in DM research. We explore the interactions between plant compounds and crucial GM homeostasis target proteins using molecular docking and other in silico approaches. Although in silico approaches have been used broadly with limitations and uncertainties, translating these *in vivo* successes into viable GM homeostasis strategies necessitates a more holistic approach beyond promising laboratory results. Our groundbreaking research unveils the remarkable GM-modulating properties of small molecules derived from MQEO. These novel molecules exert their therapeutic effects by effectively modulating the composition of Gram-positive and Gram-negative bacteria, thereby altering the gut microbiomta landscape. Additionally, they disrupt the binding of LPS to the TLR4 co-receptor signaling complex, interfering with the inflammatory cascade initiated by LPS recognition. This remarkable sighting opens up new avenues for the development of effective therapeutic strategies to tournament a wide range of TLR4-mediated inflammatory conditions. Further *in vitro* and *in vivo* studies are highly warranted to fully elucidate the mechanisms of action of these small molecules and their potential clinical applications.

## Funding statement

Not applicable.

## Institutional review board statement

Not applicable.

## Informed consent statement

Not applicable.

## Data availability statement

Data are contained within the article and the supplementary materials.

## CRediT authorship contribution statement

**Mahmoud Dahab:** Writing – original draft, Supervision, Methodology, Investigation, Formal analysis, Conceptualization. **Hajo Idris:** Writing – original draft, Validation, Software, Project administration. **Ping Zhang:** Writing – review & editing, Software, Project administration, Investigation. **Mohammed Aladhadh:** Writing – review & editing, Software, Investigation. **Eid A. Alatawi:** Writing – original draft, Project administration, Methodology, Investigation, Conceptualization. **Long Chiau Ming:** Writing – original draft, Software, Resources, Methodology, Conceptualization. **Khang Wen Goh:** Writing – review & editing, Visualization, Software, Resources. **Hooi-Leng Ser:** Writing – original draft, Project administration, Methodology, Investigation, Conceptualization.

## Declaration of competing interest

The authors declare that they have no known competing financial interests or personal relationships that could have appeared to influence the work reported in this paper.
