# Supplementary material for: Influence of Maqian essential oil on gut microbiota and immunoresponses in type 1 diabetes: In silico study
Source: Heliyon. 2024 Apr 15;10(8):e29490. doi: 10.1016/j.heliyon.2024.e29490 (PMC11035065; doi:10.1016/j.heliyon.2024.e29490)
Supplement: Multimedia component 1 [file mmc1.docx]

**Table S1. Table S1.** Binding energy (kcal/mol) of MQEO small molecules with target proteins.

| **Compound name** | **7dmq**^a^ | **3fxi**^b^ |
| --- | --- | --- |
| α-Pinene | -5.9 | -7.2 |
| β-Pinene | -6.5 | -7.2 |
| α-Phellandrene | -5.7 | -5.3 |
| 3-Carene | -5.5 | -6.4 |
| P-Cymene | -6 | -6.6 |
| D-Limonene | -6.1 | -6.8 |
| β-Phellandrene | -5.2 | -6.9 |
| cis-β-Ocimene | -4.8 | -6.5 |
| trans-β-Ocimene | -5.5 | -6.5 |
| α-Terpineol | -4.3 | -5.8 |
| n-Decanal | -4.7 | -5.5 |
| Acetic acid octyl ester | -3.5 | -5.6 |

^a^ : Cas13a anti-tag RNA ternary, *Leptotrichia shahii*, **^b^**: TLR4/MD-2.


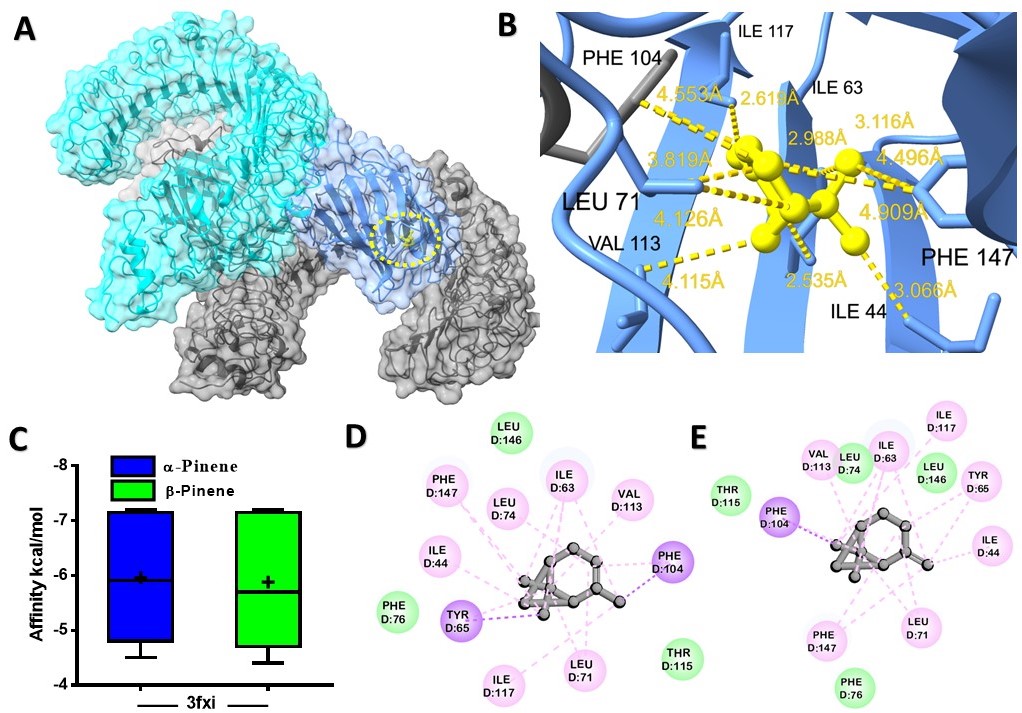


**Fig. S1.** Molecular docking analyses of α-Pinene and β-Pinene against TLR4/MD2; (**A**), Pose view of the interaction of α-Pinene and β-Pinene to the TLR4/MD2 protein, (**B**) 3D interaction of β-Pinene with TLR4/MD2, (**C**) Box plot depicted binding affinity scores for predictions of α-Pinene (blue) and β-Pinene (green) with TLR4/MD2 protein (3fxi) protein, (**D**) 2D interaction of TLR4/MD2 protein with α-Pinene. (**E**) 2D interaction of TLR4/MD2 protein with β-Pinene.


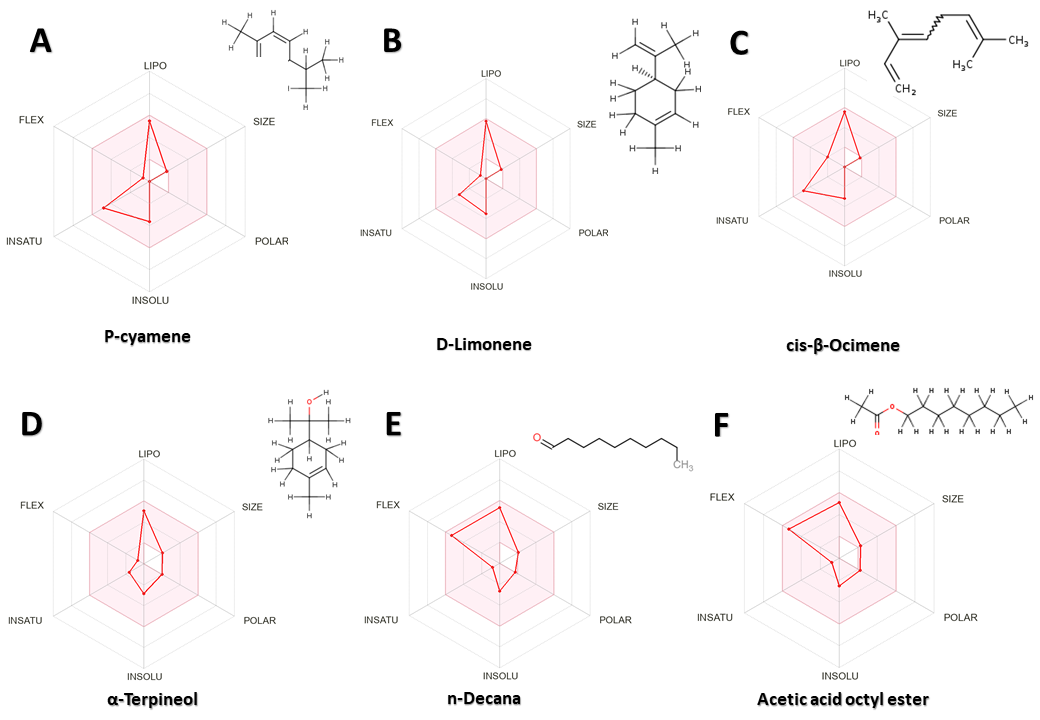


**Fig S2.** Evaluation of bioavailability of the MQEO, a small molecules modulete gut microbiota**.** The pink area of the bioavailability radar graph represents drug-likeness properties of the molecule.
